# Active Screening for Pulmonary Tuberculosis Among Jail Inmates: A Mixed Method Study From Puducherry, South India

**DOI:** 10.7759/cureus.39749

**Published:** 2023-05-30

**Authors:** Gaurang Narayan, Kavita Vasudevan, Anandaraj Rajagopal, Kalaipriya Gunasekaran, Hritik Savla, Tarun Kumar Suvvari

**Affiliations:** 1 Department of Medical Law and Ethics, National Law School of India University, Bengaluru, IND; 2 Department of Obstetrics and Gynecology, Indira Gandhi Government Medical College, Nagpur, IND; 3 Department of Community Medicine, Indira Gandhi Medical College and Research Institute, Pondicherry, IND; 4 Department of Surgery and Community Medicine, Grant Government Medical College and Sir JJ Group of Hospitals, Mumbai, IND; 5 Department of Medicine, Squad Medicine and Research (SMR), Visakhapatnam, IND; 6 Department of General Medicine, Rangaraya Medical College, Kakinada, IND

**Keywords:** cb-naat, mixed methods research, jail inmates, pulmonary tb (ptb), active case finding (acf)

## Abstract

Background: Sustainable Development Goal 3 (SDG) aims to end the epidemic of TB by 2030. To achieve this goal, active screening should be initiated in the target populations. These target populations are those without access to proper healthcare like jail inmates. With pulmonary tuberculosis (PTB) being cosmopolitan in India, passive case finding alone cannot suffice to achieve the above-mentioned goal. Thus, active case finding (ACF) becomes the need of the hour. So, we aimed to conduct a mixed methods study that has a quantitative component, i.e., to actively screen the prison inmates for PTB, and a qualitative component, i.e., to know the perceptions of jail inmates towards PTB and the stigmas associated with it.

Methodology: This was a mixed-method study conducted in the Central Jail, Puducherry. The quantitative component involved a facility-based cross-sectional study design and the qualitative component involved a focused group discussion (FGD). Participants were screened for PTB and diabetes mellitus (DM) and their anthropometry (weight, height, body mass index {BMI}, waist-to-hip ratio {WHR}) was noted. Presumptive cases were identified as those with symptoms of cough for more than two weeks with or without other concomitant symptoms. They were subjected to cartridge-based nucleic acid amplification test (CB-NAAT) assay. Data were entered in MS Excel 2017 and analyzed using SPSS version 16 (Armonk, NY: IBM Corp). For the qualitative exercise, purposive sampling with maximum variation technique was done to enroll a diverse subset of population for the FGD. Iterative analysis of the content was performed by the team to generate codes and themes.

Results: Out of all the 187 inmates screened, 10.7% were symptomatic. On CB-NAAT examination of the symptomatic inmates, none turned positive. The inmates with presumptive TB were older by age and had a higher proportion of illiteracy and existing co-morbidity (p≤0.05). While random blood sugar (RBS) levels of >140 mg/dL were recorded in 19.7% of inmates, RBS levels of >200 mg/dL considered diagnostic were noted in 5.34% of inmates. A total of 2.67% of the inmates were newly diagnosed with diabetes mellitus. The further management of the newly diagnosed inmates was taken over by the medical supervision team of the Central Jail. From the FGD, thematic manual content analysis was performed. A total of 24 codes were generated. After merging similar codes and removing duplications, the remaining 16 codes were grouped into six broad themes. Conclusions were drawn by interpretation of these themes.

Conclusion: ACF is important as it is associated with early detection and treatment. It must be done periodically. During the FGD, we came across negative ideologies and stigmas associated with PTB among jail inmates. We used the same platform to clear those ideologies and recommend frequent health education exercises even in socially ostracized communities like jail inmates.

## Introduction

According to the World Health Organization (WHO), globally 10.04 million people were diagnosed with tuberculosis (TB) and around 1.8 million died of the same in 2015 [1,2]. To curb the devastating state of the Global TB epidemic, Sustainable Development Goal 3.3 targets to end the epidemic of TB by the year 2030 [3]. WHO End TB strategy also aims to reduce the incidence by 90% by 2035 compared to 2015 [4]. To facilitate achieving these targets, the orders on Operational Research (OR) guidelines from National Tuberculosis Elimination Program (NTEP) (back then it was the Revised National Tuberculosis Control Programme {RNTCP}) make active screening/active case finding (ACF) a priority [5].

According to WHO, ACF is basically a provider-dependant initiative with the primary objective of early detection of TB cases by finding symptomatic people in specified target groups and initiating treatment promptly [3,5]. WHO defines this systematic screening as the “systematic identification of people with suspected active TB, in a predetermined target group using tests, examinations, or other procedures that can be applied rapidly” [3].

In 2013, the WHO developed guidelines for systemic screening of TB in pre-determined selected risk groups. This list also included jail inmates [6]. It was realized that, among all the risk groups, prisoners, are often neglected. Implementing screening in jail will not only benefit prisoners and prison staff but also benefit the larger community as most of the jail inmates constitute the floating population [6,7]. A disproportionately high number of prisoners come from socioeconomically disadvantaged populations with high burden of disease and poor access to medical care. Further, prison conditions can fan the spread of disease through overcrowding, poor ventilation, poor nutrition, late diagnosis, repeated prison transfers, and concomitant conditions - particularly human immunodeficiency virus infection (increased incidence of male-to-male sex {MSM} [8,9].

Cases of TB in prisons account for up to 25% of the country’s burden with a reported point prevalence of 217/1,00,000 [10]. This situation is worsened by the emergence and spread of multidrug-resistant (MDR) and extensively drug-resistant (XDR) TB [9]. The global burden of TB among prison inmates clearly indicates the need for active surveillance of TB in Indian prisons [11-14]. Studies in the Indian context also emphasize the need for active screening for TB among prison inmates [15-17]. Considering these factors, the present study aimed to estimate the burden of pulmonary tuberculosis (PTB) in jail inmates of Puducherry and identify the perceptions of the inmates towards tuberculosis.

## Materials and methods

Pre-requisites and initial arrangements

The present study involved a mixed-method study design and was conducted in Central Jail, Puducherry. It was conducted over a period of two months between September-October 2018. The study involved two phases. Phase I was the quantitative component and Phase II was the qualitative component. After obtaining due permissions from the Institute Research Committee and Institute Ethics Committee (IEC #20/140/IEC/PP/2018), permissions were sort from the concerned authorities of Central Jail, Puducherry, and the State TB office. A preliminary visit to the jail was undertaken to plan the logistics and other arrangements.

Phase I: Quantitative Component

Phase I was a facility-based cross-sectional study. All the consenting adult inmates (>18 years), excluding known cases of pulmonary or extra-pulmonary tuberculosis currently on treatment, were included in the study. The sample size included 187 jail inmates who were screened with a three-part semi-structured questionnaire (first part - sociodemographic characters, second part - signs and symptoms related to TB, third part - examination findings including anthropometric records). Anyone (presumptive TB) with a cough for more than two weeks with or without other symptoms was screened for tuberculosis [18].

A detailed history of their sociodemographic data with special emphasis on contact history for tuberculosis was obtained. They were subject to basic anthropometric measurements like weight, height, BMI, waist circumference, hip circumference, and waist-hip ratio (WHR). All the anthropometric measurements were measured using standard protocols and were compared using accepted cut-off values [19,20]. The weight was calculated using a manual adult weighing machine and the weight was measured following standard procedures with minimal clothing and without accessories and footwear. It was calculated to the nearest 0.5 kg. The height was measured using a wall sticker height chart. The subjects were asked to stand straight without their footwear, with the shoulder straight and broad, with the belly in and flat, with the buttocks and the back of thigh exactly touching the wall. The height was measured to the nearest 0.5 cm. The heights were measured in centimeters and later converted to meters by dividing the height by 100, so as to facilitate the calculation of BMI. The BMI was calculated using Quetlet's index as follows: BMI = weight in kilogram/height in meter square [19,20]. The Southeast Asian classification of BMI was used to categorize the BMI into the following various categories: underweight (<18.5 kg), normal (18-22.9 kg), overweight (23-24.9 kg), pre-obese (25-29.9 kg), obese (30-40 kg), morbidly obese (40.1-50 kg), and super obese (>50 kg).

The TB suspects were subjected to two sputum examinations by Cartridge-based nucleic acid amplification test (CB-NAAT) assay. CB-NAAT was chosen as it aids in simultaneously evaluating resistance to rifampicin by detecting the rpoB gene. The State TB Office had permitted the use of the mobile CB-NAAT testing van and on-spot testing of samples were done. The TB suspects were given falcon tubes early in the morning, with detailed instructions on the proper method of collection of the samples. The sample ID numbers, the quantity, and the quality of sputum samples were checked. Samples failing to match the requirements of 5 mL and minimal contamination with saliva were rejected and a repeat sample was asked for.

Concurrent screening of diabetes mellitus by automated glucometer was done as per RNTCP guidelines. Proper universal standard safety precautions were followed. After sterilizing the digits, the participants were subjected to capillary prick using a sterile one-time use lancet. Further, anybody who is a new case of diabetes mellitus with random blood sugar (RBS) value >140 mg/dL was advised a blood sugar examination to confirm the diagnosis. The jail doctor was also requested to follow these cases.

Data entry for the quantitative component was done using MS Excel 2017 and was analyzed using SPSS version 16 (Armonk, NY: IBM Corp). Categorical variables were expressed as proportions and continuous variables as mean with standard deviation. Student's t-test was used for comparison of means and chi-square test for comparison of proportions.

Phase II: Qualitative Component

For the FGD purposive sampling using the maximum variation technique was done. Six members, whom we believed would efficiently contribute to the richness of the data were chosen. Enrolment for the FGD was done based on their sociodemographic background and their interactiveness and receptiveness during the initial screening. Further, opinions and inputs on the participants' interpersonal skills, and cultural and environmental fitness were asked from the jail authorities. Based on the availability of logistics and convenience of each participant with regard to their availability and ongoing court sessions a preferred date was fixed. We chose a separate secluded section inside the jail premises to host the FGD and arrangements for refreshments were made.

The FGD team comprised of the authors, two social workers, and two postgraduate students of community medicine. The team was trained in conducting an FGD in the local vernacular language (Tamil) prior to the start of the session. The tools used for the FGD was a semi-qualitative FGD interview guide with 8-10 open-ended questions in it. These questions were chosen after taking into consideration the previous literature. The FGD guide was pilot tested on two members (not included in the study) and necessary modifications were made.

Care was taken to give the participants equal chances, freedom, and space to voice out their opinions without any prejudice or apprehension. The jail authorities were not present in the vicinity of the survey. The participants were clearly instructed that when one of them talks, the other listens and then adds his points or contradicts the same. One member moderated the session and three members were taking notes. All the conversations were digitally recorded and saved.

Transcripts were made from the recording and iterative analysis of the content was done. As a first step, the transcripts were reviewed for errors and omissions including context and content accuracy. Later, the content was familiarized, and after multiple readings of the text duly supplemented with the recorded audio, both inductive and deductive data were coded manually in a notebook. Initially, a total of 24 codes were generated. After merging similar codes and removing duplications, the remaining 16 codes were grouped into six broad themes. The analysis focused on themes relevant to the focus area in the framework.

Health education

Following the FGD, a talk was organized to alleviate the negative perceptions related to TB and the stigmas associated. The health talk was given to educate and create awareness among the inmates. Also, participants were requested to pass on this information to new inmates, their families, and friends. The entire survey ended with a thank you note and tea.

## Results

Quantitative component

The study population comprised 54 convicts, 37 individuals charged with civil cases (under trial one), 93 charged with criminal cases (under trial two), and three staff members. The mean age of the study population was found to be 33.72±10.38 years. The age distribution of the inmates is shown in (Table 1). The majority belonged to the age group of 26-35 years. The majority of the prison inmates were educated up to the 10th standard and have stayed in jail for less than five years (Table 2).

**Table 1:** Age distribution of the study population. *This group included three graduate professionals - engineers.

Characteristic	Distribution	Number (%)
Age group (years)	18-25	39 (20.85)
	26-35	43 (22.99)
	36-45	32 (17.11)
	46-55	29 (15.51)
	56-65	27 (14.44)
	>65	7 (3.74)
	Total	187 (100.0)
Educational status	Illiterate	18 (9.62)
	Primary school	26 (13.90)
	Middle school	52 (27.80)
	High school	47 (25.13)
	Higher secondary	16 (8.6)
	Graduate*	26 (14.0)
	Postgraduate	2 (1.1)
	Total	187

**Table 2:** Duration of stay and status in jail.

Variables	Category	Number (%)
Status of the inmates based on court trails	Convict	54 (28.9)
	Under trial-1	37 (19.8)
	Under trail-2	93 (49.7)
	Staff	3 (1.6)
Duration of stay in jail (in years)	<5	152 (81.6)
	5-10	17 (9.2)
	11-15	13 (6.9)
	16-20	3 (1.7)
	21-25	1 (0.6)

**Figure 1:**
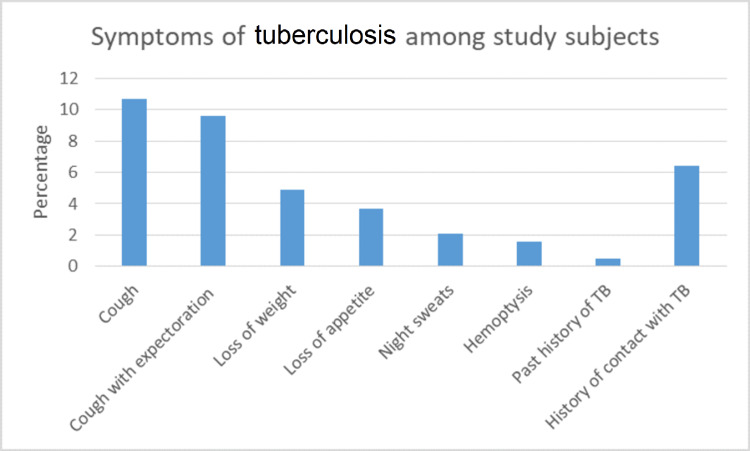
Symptoms of tuberculosis among study subjects.

About 9.6% of the prison inmates reported suffering from chronic illness like diabetes mellitus, hypertension, chronic obstructive pulmonary disorder (COPD), skin infections, and history of having been treated for tuberculosis in the last one year (Table 3). Figure 1 shows the clinical symptoms of tuberculosis among the study subjects. The proportion of presumptive TB cases among the inmates was found to be 10.7%. About 6.4% of the inmates gave a history of having been in contact with known cases of tuberculosis. The anthropometric measurements and physical findings of the presumptive TB cases in comparison to the other inmates in-toto is shown in (Table 4).

**Table 3:** Chronic illnesses in the study subjects. *Multiple responses obtained. COPD: chronic obstructive pulmonary disorder

Chronic illness present	Type of chronic illness	Number (%)
Yes (n=18)	Diabetes mellitus	12 (6.4)
	Hypertension	4 (2.1)
	COPD	2 (1.1)
	Skin illnesses	1 (0.5)
	TB	1 (0.5)
No	-	171 (91.4)
Total		189*

**Table 4:** Anthropometric measurements and physical findings in study subjects. SBP: systolic blood pressure; DBP: diastolic blood pressure; RBS: random blood sugar; TB: tuberculosis

Measurement	Total study population	Presumptive TB
Mean weight	69.06±13.10	72.9±15.64
Mean BMI	24.16±4.38	25.45±4.7
Mean waist circumference	93.63±8.23	97.65±10.1
Mean waist-hip ratio	1.07±0.06	1.05±0.5
Mean SBP	116.02±13.82	115.60±13.64
Mean DBP	65.63±9.78	70.70±9.28
Mean RBS	119.87±50.77	111.5±25.2
Mean age	33.72±10.38	39.10±13.9

Table 5 compares a few variables between presumptive TB cases and the other inmates. The inmates with presumptive TB were found to be older, had a higher proportion of illiteracy, and had a higher proportion of co-existing morbidity (p≤0.05). Of the 20 inmates with presumptive TB, four had dry cough, two were released on bail and of the remaining, only 10 people had given the samples that matched the quality for CB-NAAT examination, of which none were positive for *Mycobacterium tuberculosis*. Concurrent screening of diabetes mellitus was done of which abnormally high blood sugar of >140 mg/dL was found to be in 19.7% of the inmates and values >200 mg/dL were found in 5.34% of the inmates. Since values >200 mg/dL by RBS examination are confirmatory for diabetes mellitus, these inmates have been advised lifestyle modifications and dietary patterns change. The jail doctor has been requested to treat, monitor, and follow them up. For cases of >140 mg/dL, they have been advised to take a blood sugar examination for confirmation.

**Table 5:** Comparison of a few variables between presumptive TB and others. *Parameter being statistically significant. TB: tuberculosis; H/O: history of

Variables	Presumptive TB (n=20)	Others (n=167)
Mean age	39.10±13.9	33.07±9.72
Illiterate*	5 (25.0)	13 (7.78)
H/O contact with TB case	1 (5.0)	11 (6.5)
Past H/O TB	1 (5.0)	0
Overweight/obesity	12 (60.0)	105 (62.8)
H/O concurrent morbidity	3 (15.0)	15 (8.9)
H/O current diabetes	1 (5.0)	11 (6.5)
Abnormal random blood sugar	1 (5.0)	22 (13.2)

Qualitative component

**Figure 2:**
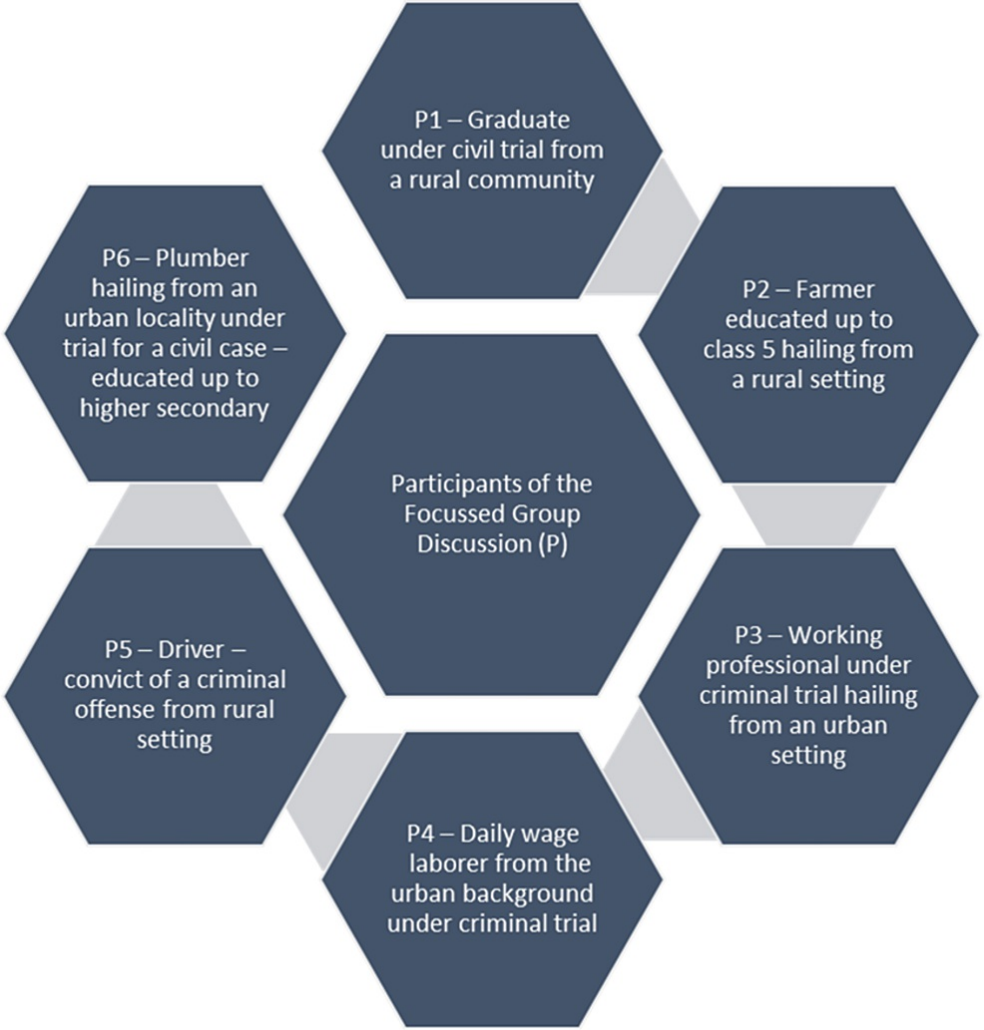
Characteristics of the participants recruited for the FGD (n=7) FGD: focused group discussion

Figure 2 highlights the characteristics of the participants included for the FGD. On iterative manual content analysis, a total of 32 codes were generated. After deleting duplicates, 28 codes were generated which were grouped into six broad themes. The six themes include mode of infection of TB, risk factors and risk groups associated with TB, symptomatology of TB, treatment perspectives of TB and its cure, impact of TB on one’s life, and prevention aspects related to TB.

Mode of TB Infection

Most of the respondents were unaware of the fact that TB is caused by a microbe. While a few of them believed that TB could be spreading through droplets, they believed TB was caused due to a fungal infection.

One of the respondents P4 stated that “Mosquito bites cause TB. It is prevalent common in rainy weather and spreads easily from one person to another.” Participant P6 added, “It spreads through DNA genes. My brother had TB, my sister is also a case of TB. It is because of my dad’s genes.”

This clearly indicates the dearth of knowledge among common people regarding TB. Though, they realize TB to be an infection and that it is communicable from person-to-person, the scientific reasoning behind this is commonly misunderstood by them. While genes do have a possible role to play in the development of resistance to drugs and the type of immune response mounted by different individuals, recognizing overcrowding and person-to-person transmission by poor respiratory hygiene is the key to adopting preventive behavior. Delivering these facts to the common public is important.

Risk Factors and Risk Groups Associated With TB

According to the group, smoking, spitting saliva, sneezing, pollution, and eating chilled items were believed to be risk factors for TB infection.

Participant P5 added that “TB usually develops following rainy season, drinking cold items like lassi. It usually affects group of people from same village who do not complete the yearly religious rituals.”

Attaching religious and cultural practices to the development of disease clearly indicates the cultural diversity that is commonly prevalent across communities in India. Thus, devising educative models that would receive cultural acceptance becomes important to improvise and strengthen the healthcare-seeking behavior of individuals.

One participant P3 after patiently listening to the initial conversations stated that “When our defense system fails, we can develop TB. When our white blood cell (WBC) counts fall, we develop TB. After treatment of cancer, WBCs fall. Also, TB does not spread through genes, it is because of close contact.”

These words coming from a postgraduate ex-working professional in an urban setting clearly indicate the impact of proper education and social upbringing on knowledge and healthcare-seeking/preventive behaviors. This also validates the results obtained in the quantitative component. Higher rates of illiteracy are associated with poor preventive behavior and may pose a threat to contracting TB.

Symptomatology of TB Infection

Most of the respondents believed TB to present with cough (usually dry), fever, and weight loss. When probed further, a few added symptoms like fatigue, giddiness, shivering, chills, and swelling of joints.

Participant P2 stated that “One of my neighbors had developed TB. Cough and weight loss was his main complaint. He was suffering for a long time and over time he became completely frail.”

There seemed to be consensus on the symptomatology and presentation of symptoms with regard to TB across all participants. This indicates the fact that despite hailing from different educational and societal backgrounds, experiences with patients, relatives, and friends had left an impact on the common man. This indirectly reflects the burden of TB in India.

Treatment Perspectives of TB and Its Cure

All the respondents knew that TB was curable with specific treatment. Most of them believed that an early diagnosis would cure TB completely and prevent TB from affecting other organs/organ systems in the body. Though, they were all aware that the treatment for TB was available across all government-run hospitals, not many respondents knew the course/duration of anti-tubercular therapy (ATT). It was interesting to note that the respondents believed that they knew certain side effects of the drugs. They also mutually agreed that if left untreated, TB could derange the body's functions and eventually cause death.

One of the respondents P1 shared his personal experience and stated that “My brother-in-law was affected with TB 1.5 years back and was provided treatment at a government chest clinic opposite to Jawaharlal Institute of Postgraduate Medical Education and Research (JIPMER). He was given tablets for six months. If medicines are taken regularly, the cure is a sure thing. He was given daily medicines and injections.”

To this respondent P4 added, “Treatment purely depends on the stage of disease. I had a friend who was critical. The medicines damaged his kidney and nerves.” Additionally, one participant P5 said, “Certain food like palmyra must be avoided during treatment with medicines.”

One of the participants P6 stated that “The treatment is available in all public district hospitals and the government is taking sterner actions to cut down TB infection.” One of the respondents P3 supplemented the previous statement. He added that the drugs were also provided by a few NGOs and stated that “There are few non-governmental organizations (NGOs) like a team at Rowthamkuppam, a village in Pondicherry, they used to select such TB infected cases and provide them with free treatment.”

In opposition to the previous statements, one participant P2 responded, “Treatment is free of cost in government sectors, but people feel government treatments are cheap and not up to the mark. So, most of my relatives and people in my village prefer treatment only in private hospitals.”

Though awareness regarding the treatment, options, and adverse effects was noted among the participants, prejudice to the existing public healthcare delivery models is a matter of concern.

Impact of TB on One’s Life

This segment explored the hidden myriad facets of sociocultural stigmas prevalent in the community towards TB patients. People lose jobs and they end up in debt. They are ostracized by the society.

One of the participants P5 stated that “After a patient develops TB, he must stay home and isolated from family. There is a lot of financial burden.” To this statement, respondent P6 added, “Mental health of an individual is more affected than physical. More than others avoiding us, we ourselves avoid mingling with others, as we do not want our kids and wives to contract TB. Rather than society isolating, the person himself distances.”

Participant P1 supported the statements of P6 and added, “The mental health of the patient is first affected which then channels as bad mood to all other people. TB by itself is disastrous and is a repercussion of the sins we did.” Participant P2 stated that “People do not prefer to share food with the affected individuals.”

When the interviewer probed further into other issues/constraints, P4 added, “People prefer private clinics and thus end up paying more.” He further added, “Individual attention is not given in GH like private clinics. When it comes to our family, we don’t want to take that call. We take them to private set-ups and land in debt.”

From one side of the table, there was a realization among the respondents that physical isolation and fear of spreading the infection to close family members and friends affects mental health of the affected individuals; from the other side of the table, this realization was noted only among participants from working background. Among the respondents, financial constraints were identified as a concern more than one’s mental health and emotional well-being.

Further, perceptions of people towards the quality of healthcare delivery in public institutions impede outreach of the available healthcare programs. Logistics, manpower, funding, public relations, and outreach are areas that require strengthening for effective deliverables to community health.

Prevention Aspects Related to TB

Participants believed that cleanliness and practicing yoga could avert TB infection. They added masks can be used to avoid infection.

Participant P4 added that “There has to be strict handwashing before and after food.” Participant P1 emphasized, “It is important to maintain distance from patients.” Another respondent P2 added that “Every six months or yearly once, there must be a voluntary full body check-up of individuals and families.”

When the interviewer probed him about specific target groups for screening, he mentioned, “If the disease is prevailing in family or heritage, the family members needed to get themselves checked. If many from the same community are affected then the entire community should be screened.” Only two respondents were aware of TB vaccine. Participant P6 stated that “I read in my son’s book that there is a vaccine to avoid TB. It can be given up to five years and there are 18 such injections.”

It was evident that the cohort acknowledged the importance of cleanliness, hygiene, social distancing, and avoidance of overcrowding with preventive behavior. Yet, knowledge of routine screening and availability of vaccines was poor and they point to the need for regular community health education.

## Discussion

In the present study, a total of 187 prison inmates of Central Jail in Pondicherry were screened for tuberculosis, of which 10.7% were symptomatic for tuberculosis. These inmates were subjected to CB-NAAT examination and none were positive for *Mycobacterium tuberculosis*. This result may be due to the small sample size of the study population and due to security issues, we were not permitted to follow-up on the 10 participants of presumptive TB who could not give a sample.

Throwing light on the global scenario, the study conducted in the prisons of Ethiopia by Adane et al. screened the inmates of the prison using sputum compound light microscopy. The estimated point prevalence of total undiagnosed TB cases was found to be 505/100,000 prisoners. The total population study size was 112,361, of which the inmates with symptoms were around 810. So, the percentage of symptomatic participants is only 0.72% which is comparatively lesser than our results. The reason behind not detecting a case of tuberculosis through active screening could be attributed to the larger sample size of the prison in Ethiopia and the presence of overcrowding among the individual cells of the jails of Ethiopia which are stuffed with more than six inmates per room. Also, they used sputum light microscopy which can detect positive cases only when the bacterial load is high, in the order of 10³. While in our study, we used CB-NAAT which can pick up cases even in the presence of less load, as well as simultaneously detect resistance to any drugs, if any [11].

Similar differences are explained even in the study conducted by Fuge and Ayanto in the prison of Hadiya in Southern Ethiopia. There were total 809 prison inmates screened, of which 164 (20.27%) were symptomatic and the point prevalence of smear-positive pulmonary tuberculosis was found to be 349.2/100,000 population [12].

In the study conducted by Saunders et al., in federal prisons, a tuberculin skin test was used for active screening of tuberculosis. The study suggested improvised results in treatment outcomes because of early detection. We have not chosen a tuberculin skin test as in India the disease burden is higher than in the Federal States, as well there is a chance to miss on people who may show negative tuberculin test but are disease positive, like immunocompromised people, prior BCG vaccination [13]. The study conducted by Khaing et al. in Myanmar seconds the finding of Saunders et al., It concluded that ACF can provide improved treatment outcomes in patients [14].

To highlight the studies performed in India, the study conducted by Mallick et al. screened 16,934 inmates, of which 1348 inmates fell into the category of presumptive TB. The symptoms were seen in around 7.96% of the study population which is lesser in comparison to our study. The number of detected cases by active case finding using sputum microscopy was found to be 124 which accounts for 9.19% of the symptomatic cases. These differences can be explained based on the following important distinctions [9].

The study by Mallick et al. included a large sample size and the survey population hailed from the northern states of India with a high burden of TB. Also, the mean duration of stay of most participants in the present study was less than five years. Further, the quality of the environment inside the jail in Puducherry was known to be better than other jails in addition to providing adequate nutritional support to the jail inmates [9]. The other studies done among prison inmates in Prisons of Belgaum, Karnataka and Pune, Maharashtra have reported among other morbidities, a prevalence of TB ranging from 4.3% to 7.5% which is lesser than the prevalence of symptomatic cases in our present study [16,17]. Perspectives of inmates, as gathered from the FGD, are addressed in (Table 6).

**Table 6:** Summarized results of FGD bringing out the positive and negative perceptions of the participants towards TB. FGD: focused group discussion; TB: tuberculosis; DOTS: directly observed treatment short course

Positive	Negative
TB is curable	TB is a cancer
Few inmates were aware of the injectable drugs and DOTS providers	Causative agent of TB is a fungi and virus and has genetic background
They knew it spreads through respiratory route	Spreads through mosquito bite and spitting of saliva
They were well aware of the symptoms of TB, such as cough, weight loss, and fever. They are aware of smoking being a risk factor for TB. One of them knew it spreads through close contacts	They think the cough is dry cough and they relate contracting TB with consumption of buttermilk, lassi, and palmyra fruit
They are aware that TB is curable	A few of them feel it is a sin of the past
They are aware of various treatment providers and the pros and cons associated with the options	They agree to the social stigmas of TB like ostracizing of patients
They are well abreast of the side effect of drugs and about use of personal protective equipments (PPEs) (masks) and vaccinations available for children in relation to TB	Many prefer private sector than government because of inadequate personal patient-to-patient care in public setup

Strengths and limitations of the study

One of the biggest strengths of the study is the study setting. While ACF is receiving priority over the years, there are very few studies that screen jail inmates. Unfortunately, jail inmates are still viewed through the lens of suspicions and are subjected to stigma. Despite not being able to detect a sputum-positive case of tuberculosis, the qualitative component helped us identify the raw areas that require strengthening. Using the same platform for educating the socially ostracized community helped take a first step towards creating a change.

Due to logistic and permission issues, we were not able to follow-up on the cases of presumptive TB who were released on bail due to security concerns. In the future, we plan on conducting frequent screening programs for the inmates and this time plan to include the screening for HIV/AIDS and other sexually transmitted infections (STIs) in addition to TB and DM.

## Conclusions

Active screening should be undertaken on a periodic basis to improve case detection and thus, indirectly facilitate the elimination of TB by 2030. It will help in detecting the cases early, thereby helping to initiate treatment early and also prevent catastrophic costs, to be concerned with the treatment, economic rehabilitation, and preventing drug resistance. This opportunity can also be used for screening other co-morbidities like diabetes mellitus, hypertension, and HIV.

Periodic sessions of awareness should be hosted for the floating population who are under trial, as they have the chance to transfer the infection from the general community to the inmates of jail as well as transfer the infection from the inmates to the community. Creating awareness in such population would prove helpful as this cohort would share the message with the new inmates and the general public once they go out. Further sensitizing the jail wardens and other police staff regarding the risk factors and symptomatology of tuberculosis becomes important.
